# Patient-specific modeling for guided rehabilitation of stroke patients: the BrainX3 use-case

**DOI:** 10.3389/fneur.2023.1279875

**Published:** 2023-11-30

**Authors:** Vivek Sharma, Francisco Páscoa dos Santos, Paul F. M. J. Verschure

**Affiliations:** ^1^Donders Institute for Brain, Cognition and Behavior, Radboud University, Nijmegen, Netherlands; ^2^Eodyne Systems S.L., Barcelona, Spain; ^3^Department of Information and Communication Technologies, Universitat Pompeu Fabra (UPF), Barcelona, Spain

**Keywords:** automatic lesion identification, whole-brain models, transcranial ultrasound stimulation, BrainX3, stroke

## Abstract

BrainX3 is an interactive neuroinformatics platform that has been thoughtfully designed to support neuroscientists and clinicians with the visualization, analysis, and simulation of human neuroimaging, electrophysiological data, and brain models. The platform is intended to facilitate research and clinical use cases, with a focus on personalized medicine diagnostics, prognostics, and intervention decisions. BrainX3 is designed to provide an intuitive user experience and is equipped to handle different data types and 3D visualizations. To enhance patient-based analysis, and in keeping with the principles of personalized medicine, we propose a framework that can assist clinicians in identifying lesions and making patient-specific intervention decisions. To this end, we are developing an AI-based model for lesion identification, along with a mapping of tract information. By leveraging the patient's lesion information, we can gain valuable insights into the structural damage caused by the lesion. Furthermore, constraining whole-brain models with patient-specific disconnection masks can allow for the detection of mesoscale excitatory-inhibitory imbalances that cause disruptions in macroscale network properties. Finally, such information has the potential to guide neuromodulation approaches, assisting in the choice of candidate targets for stimulation techniques such as Transcranial Ultrasound Stimulation (TUS), which modulate E-I balance, potentiating cortical reorganization and the restoration of the dynamics and functionality disrupted due to the lesion.

## Introduction

Stroke, being the second leading cause of disability and death on a global scale, poses a considerable burden to individuals and society alike (1). The staggering number of disability-adjusted life-years (DALYs) lost, ~116 million in 2016 alone, underscores the urgent need for effective stroke management (2). Despite some improvements in age-standardized mortality rates for ischemic stroke (IS) and hemorrhagic stroke (HS) from 1990 to 2010, there was a substantial increase in the absolute number of incident IS and HS cases by 37 and 47%, respectively (3). Given statistics, the importance of rehabilitation in stroke care cannot be overstated. Effective rehabilitation interventions play a crucial role in minimizing disability, optimizing recovery, and improving the quality of life for stroke survivors. Early and tailored rehabilitation efforts are essential to address the diverse physical, cognitive, and emotional challenges faced by stroke patients, ultimately empowering them to regain independence and participate fully in their daily lives.

The decreased quality of life in stroke patients is a result of the associated symptoms which extend beyond the well-known motor impairments, to the domains of sensory processing and higher-order cognition (4–6). In addition, stroke-related side effects such as depression and chronic pain can also have a significant impact on a patient's quality of life (7, 8). While some of these symptoms can be directly attributed to the loss of lesioned areas, post-stroke functional disruptions spread beyond the lesioned area to the rest of the brain, a phenomenon known as diaschisis (9). In fact, stroke lesions have been shown to lead to diverse changes in cortical functional connectivity and its network properties (9, 10), which often correlate with some of the pathological deficits observed in stroke patients (11, 12). Importantly, these are not limited to edge-specific changes in FC and disruptions in complex graph properties of functional networks have been related to stroke deficits and progression. More specifically, lesions in connector hubs (i.e., nodes connecting functional modules), as opposed to lesions in sub-network hubs affect the integrity of functional network organization (13) through decreases in both local (14) and global (14, 15) graph efficiency. In addition, studies also show a decoupling between structural and functional connectivity, correlated with motor function (15). On another note, modularity, quantifying the balance between the integration and segregation of functional networks (16), is also affected in stroke patients. Importantly, modularity evolves toward healthy levels over a time scale of months, relating to the recovery of higher-order cognitive functions such as memory and language (16). All in all, the literature points out connectional diaschisis as a pivotal phenomenon in stroke patients, pushing the focus away from focal deficits caused by gray matter loss toward network dysfunction (9).

Perhaps more intriguing than the phenomenon of diaschisis itself is the fact that cortical networks, being highly plastic, particularly during stroke recovery (17), can reorganize to recover abstract properties of FC such as modularity toward pre-lesion levels (18). In that regard, recent work suggests that the recovery of local balance during the months following a stroke may not only be a key piece of recovery (17, 19) but also underlie the re-emergence of disrupted properties of functional networks in the neocortex, such as modularity (18, 20). This view is strongly supported by literature, which indicates a relationship between stroke recovery and persistent increases in local excitability (21–29). Therefore, we suggest that the framework of mesoscale E-I homeostasis and its impact on macroscale properties of functional networks is essential in understanding, and thus potentiating, the process of stroke recovery. Under this framework, large-scale models of the human brain provide a useful tool for the study of how local E-I balance shapes macroscale dynamics and networks, given that changes in excitability are difficult to probe non-invasively (18, 20). Therefore, we argue that whole-brain models, constrained by patient-specific lesion information and accounting for local homeostasis can be a powerful tool to assess the critical changes required to recover local balance and, consequently, promote the reorganization of large-scale networks.

**Figure 1:**
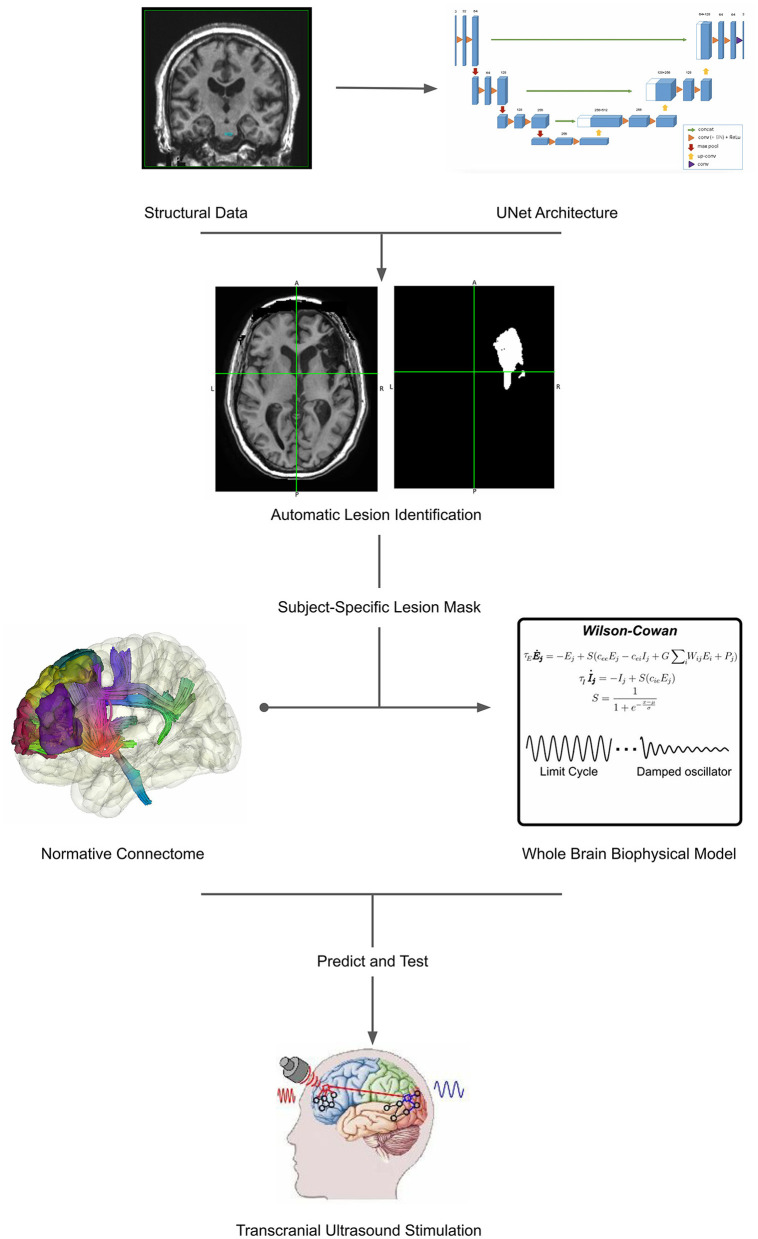
Workflow illustrating the comprehensive approach proposed in the article. The process begins with automatic lesion detection (30), which generates a lesion mask from neuroimaging data (31). This lesion mask serves as a foundation for patient-specific modeling, facilitating the creation of personalized brain models. These models encompass both structural and functional aspects, providing a holistic representation of the individual's brain. Subsequently, non-invasive transcranial ultrasound stimulation (TUS) (32) is applied to target specific regions for neurorehabilitation. This integration of lesion detection, patient-specific modeling, and TUS presents a cohesive framework for advancing personalized medicine in neurological care. The representative Unet architecture image, whole brain biophysical model and transcranial ultrasound stimulation are adapted from Ronneberger et al. (30), Pathak et al. (33), and Transcranial Ultrasound Stimulation and Its Applications in Neurosciences, (32) respectively.

Therefore, to enhance the prognostic ability of current clinical practices, new pipelines should be developed to account for patient-specific mapping of lesioned networks, together with models that allow not only the prediction of patient recovery but also the testing of neuromodulation approaches. For that reason, in this article, we introduce a pipeline for lesion detection and patient-specific whole-brain modeling within the versatile framework of BrainX3. As a cutting-edge neuroinformatics platform, BrainX3 offers a promising avenue for integrating these methodologies seamlessly (Figure 1).

In BrainX3 (34), we foresee a transformative integration of AI-based lesion identification and whole-brain models, ushering in a new era of personalized medicine. More specifically, through accurate lesion detection, classification, and subsequent integration in whole-brain models, this pipeline enables a dynamic understanding of neural interactions, paving the way for enhanced diagnosis and prognostic insights. Leveraging this holistic knowledge, BrainX3 can help to identify potential targets for non-invasive neuromodulation techniques like Transcranial magnetic stimulation (TMS), transcranial direct current stimulation (tDCS), and transcranial ultrasound stimulation (TUS), revolutionizing intervention strategies with personalized precision.

In the following sections, we discuss current techniques for automatic lesion segmentations, state-of-the-art large-scale modeling studies and the key aspects of their application to the context of stroke and the potential of techniques such as TMS for targeted stimulation of key areas for stroke recovery. Finally, we present a unifying framework, within BrainX3, for the seamless integration of all these steps in a pipeline for clinical use.

## Automatic lesion segmentation

Medical imaging techniques, such as Computed Tomography (CT), CT perfusion (CTP), Positron Emission Tomography (PET), and Magnetic Resonance Imaging (MRI), have shown great promise in providing essential information about stroke lesions' shape, size, location, and metabolism. Utilizing these imaging modalities can aid clinicians in increasing the survival rate of patients affected by ischemic stroke. Among these techniques, Magnetic Resonance Imaging (MRI) is preferred due to its sensitivity to early parenchymal changes of infarction (35).

Numerous biomedical image segmentation methods based on thresholding, region growing, statistical pattern recognition, fuzzy modeling, and Markov random field techniques have been explored in recent literature (36–38). While some methods require user interaction and are not fully automated, many automatic segmentation approaches rely on hand-designed features (39, 40). These techniques may produce erroneous results when dealing with small lesions or lesions in close proximity to normal tissue. To address these challenges, the application of Convolutional Neural Networks (CNNs) such as U-Net has gained significant interest for image classification and segmentation in various fields, including stroke lesion segmentation from multi-modal MRI (30, 41–45). U-Net architecture involves incorporating a substantial number of feature channels during the upsampling phase. This allows the network to propagate context information to higher resolution layers, resulting in a U-shaped architecture with the expansive path mirroring the contracting path. Notably, the network does not include any fully connected layers and solely utilizes the valid part of each convolution. Consequently, the segmentation map contains only the pixels with complete context available in the input image. This strategic approach enables the smooth segmentation of arbitrarily large images through an overlap-tile strategy (30).

The adoption of the U-Net architecture can play a pivotal role in facilitating the identification of lesion sites, which holds immense significance in the analysis of patient-specific disruptions in structural connectivity and simulation of whole brain activity. Therefore, more accurate lesion segmentation enables clinicians and neuroscientists to precisely pinpoint areas of structural damage caused by the lesion. Consequently, when combined with other neuroinformatics tools, such as BrainX3 (46), the U-Net-based lesion identification can provide valuable insights into the patient's connectome and contribute to a comprehensive understanding of irregularities in large-scale brain dynamics. At SPECS lab we are working on training a U-Net architecture on a publicly available database for the identification of lesions and further solidifying the BrainX3 clinical use case.

## Computational modeling for stroke

Since the dawn of connectomics (47, 48) estimates of white-matter structural connectivity of the human cortex can be obtained from MRI recordings and used to constrain models of interacting populations. The dynamics of such populations can be abstracted using mean-field models, which allow for the modeling of large-scale dynamics at a higher level of abstraction which is more computationally viable and tractable (49). Since the study by Honey and colleagues (50) macaque structural connectivity, large-scale models of the brain have been used to elucidate the relationship between structure and functions and how properties such as conduction delays, local excitatory-inhibitory (E-I) balance and oscillatory dynamics underlie proper cortical function (51–56).

Such approaches have also proven useful in the context of stroke. The first model of the effect of localized lesions on the macaque cortex showed that lesions to structural hubs of the connectome lead to larger changes in system dynamics, spread even beyond the vicinity of lesions, akin to the phenomenon of diaschisis (57). Ever since further stroke models have elaborated on the fragility of subnetworks to structural damage (58) and how disconnection affects the graph properties of FC (59).

Furthermore, by relying on local interactions between excitatory and inhibitory populations, approaches such as the Wilson-Cowan model (60) allow for the analysis of cortical microcircuitry at the mesoscale level (e.g., E-I coupling). Indeed, recent results suggest that FC of stroke patients can be optimally fitted when local inhibition is decreased, compared to healthy controls (61), in line with previous results (24) supporting the participation of E-I homeostasis in recovery (18).

Furthermore, due to the plastic nature of cortical networks and the evidence of relevant plasticity occurring after stroke (17, 19, 62), particularly at the level of E-I homeostasis (63), it is relevant to account for such mechanisms in whole-brain models of stroke. Vattikonda et al. (64) were the first to demonstrate that E-I homeostasis was able to return resting-state FC close to pre-lesion levels and that the extent of recovery correlated with the graph properties of lesioned areas. Further studies with other homeostatic mechanisms have shown their importance for not only FC but also network dynamics (65). Recently, the work of Páscoa dos Santos et al. (18, 20), extended these previous approaches by showing that, by accounting for local E-I homeostasis, models could not only explain the re-emergence of network properties such as modularity but that the distribution of changes in local excitability explains empirical findings and might relate to the emergence of late-onset side effects such as depression. In parallel, Chakraborty et al. (66) have used a similar approach to investigate the key areas for the recovery of E-I balance after stroke in order to promote the restoration of functional networks.

Therefore, mean-field based large-scale models are a powerful tool for probing into the influence of mesoscale dynamics on the macroscale organization of the neocortex, with implications for functional recovery in stroke patients, particularly when E-I homeostasis is accounted for.

In the case of stroke, being able to study these processes in personalized models is of particular importance. More specifically, models accounting for local E-I homeostasis can allow clinicians to predict the magnitude of adaptation in local excitability required for the reorganization of large-scale functional networks (20). Such knowledge can then be used in the clinic to guide neurorehabilitation or neurostimulation therapies toward optimal targeting of brain areas that might be pivotal for optimal recovery (67).

Subject-specific information is pivotal for personalized models, both at the level of gray-matter necrosis and white-matter disconnection (68, 69). While it is possible to constrain models with individualized estimates of SC (65), such data generally does not attain a satisfactory level of signal-to-noise reaction and results indicate that the use of individualized connectomes is not the most critical step in building patient-specific models (70). To that effect, normative connectomes from open databases, such as the HCP (71), can be constrained by subject-specific lesions, obtained through either manual or automatic segmentation of structural T1 MRI imaging (30). This approach has proven successful in whole-brain modeling (72), where lesion masks were used in the healthy connectome to reproduce post-stroke disruptions in functional networks.

Finally accounting for E-I homeostasis might provide key insights into the evolution of brain networks in the months following the stroke (20). Firstly, by simulating healthy activity in models constrained by a normative connectome, and applying patient-specific lesions to these healthy models, one can predict the regions more impacted by the loss of incoming excitation from the lesioned area. Most importantly, these lesioned models can provide a picture of the evolution of local excitability that would allow for the optimal reorganization of functional networks, which can be compared with the actual progression of stroke patients. More specifically, models with the same large-scale anatomical constraints and patient-specific lesions can be fitted to empirical data (e.g., fMRI) obtained along the progression of the disease by optimizing the weights of local excitatory-inhibitory coupling of the neural masses. These values can then be compared to the predictions obtained from models with fully operational E-I homeostasis, to aid in the identification of critical targets for neurostimulation to promote recovery of balance in regions where E-I homeostasis might be unable to do so (20). With that in mind, we advance this multiscale approach as a potential framework for providing valuable spatially specific information to guide clinician decisions on rehabilitation protocols, promoting optimized recovery.

## Non-invasive neuromodulation

After identifying the lesion site and potential target areas for non-invasive stimulation using whole brain models, various non-invasive neuromodulation techniques can be explored for their application in clinical settings to promote neural plasticity and enhance function in stroke patients. Transcranial magnetic stimulation (TMS) and transcranial direct current stimulation (tDCS) offer different effects on neurons, with TMS inducing neuronal depolarization through a magnetic field, while tDCS modulates cortical excitability through hyperpolarization or depolarization of neuronal resting membrane potentials (73–79). However, limitations such as spatial resolution and penetration depth have been observed in these techniques, hindering their full potential for stroke treatment (73). On the other hand, transcranial-focused ultrasound stimulation (TFUS) emerges as a promising non-invasive, high-resolution, and safe technology that can effectively modulate neural activity and exert neuroprotective effects (80, 81). Studies have demonstrated its efficacy in treating various neurological disorders, including stroke, without causing tissue damage. With its potential to become a non-invasive treatment method for ischemic stroke, transcranial ultrasound stimulation (TUS) holds promising research prospects and offers a valuable avenue for patient-specific intervention decisions in stroke management (82–85).

The use of focused ultrasound has witnessed a surge in various applications, with diagnostic ultrasound becoming an essential clinical imaging modality (86–88). Transcranial-focused ultrasound (TFUS) is a promising non-invasive technology, that offers capabilities to monitor cerebral circulation with high temporal and spatial resolution (89). TFUS allows precise delivery of energy to brain tissue at various intensities, enabling modulation of nervous system activity through frequency, intensity, and stimulation time adjustments (90). This stimulation involves transmitting ultrasound waves in continuous or pulsed forms via an ultrasonic probe (67, 91, 92). Importantly, TFUS can modulate neural excitability (93) through mechanical effects (94), alterations in ion channels (95) and membrane capacitance (96), generation of sonopores (97), and thermal effects through temperature elevation caused by sound waves (93, 98). As a potential non-invasive treatment for neurological diseases, TFUS's therapeutic effect is influenced by factors like carrier frequency, peak intensity, duration, pulse repetition frequency, and duty cycle (91). Utilizing ultrasound phased array technology, TFUS allows large-scale neuromodulation within a tissue volume by directing focused ultrasound beams to different neural targets (99, 100). Alternatively, single-element transducers enable targeted transmission of acoustic energy to specific areas in the brain, acting on focal points (101). Low-intensity focused ultrasound (LIFU), operating at specific frequencies and intensities stimulates nerve tissue mainly through pressure generated by ultrasonic radiation (92). LIFU can improve blood supply around brain lesions via neural regulation without causing tissue damage, making it a promising option for the non-invasive treatment of ischemic stroke (102–104).

Therefore, given the potential of ultrasound stimulation techniques to modulate neural excitability or improve blood supply around necrotic areas, we highlight their potential to supplement current rehabilitation therapies and potentiate recovery post-stroke.

## BrainX3-use case

**Figure 2:**
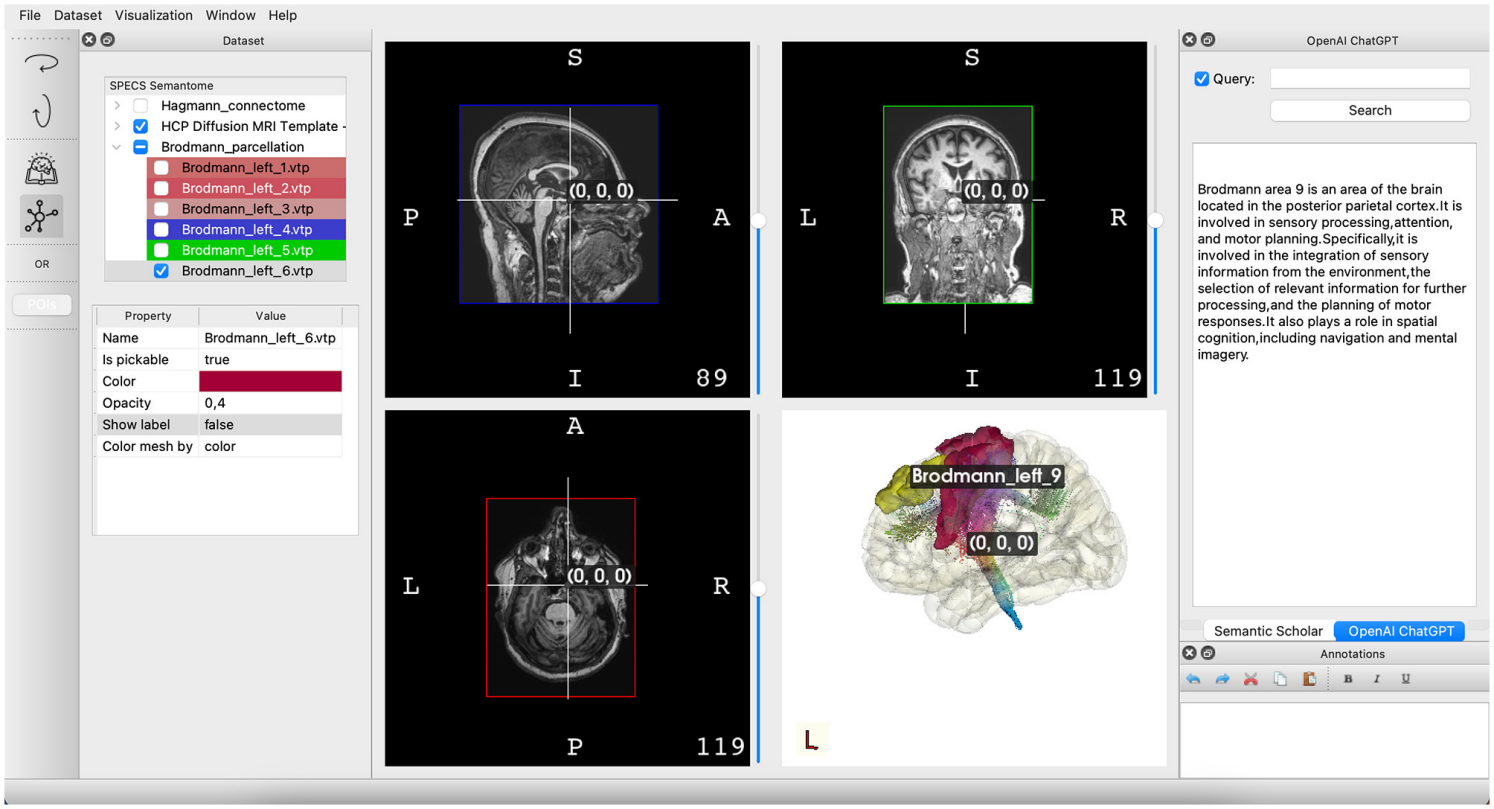
Illustration of the primary interface in BrainX3 2.0. On the **left** pane, you'll find showcased datasets, encompassing atlases and imaging information. The **center** pane is designated for the visualization of imaging data and a three-dimensional portrayal of the brain structure. Lastly, the **right** pane features the BrainX3 Semantome, employing API-driven search tools to retrieve up-to-date research articles and leveraging OpenAI's chat GPT to respond to inquiries.

BrainX3 serves as an exemplary platform for personalized medicine, developed at SPECS lab where the team is currently working on integrating lesion segmentation, whole-brain modeling, and non-invasive stimulation protocols. It adopts a layered architecture with distinct levels: Graphical User Interface (GUI), Application Core, Native, and Specific Plugins. This design ensures decoupling of the user interface from the internal application logic, as well as the internal logic from the data types and components. The GUI layer handles user interfaces and interactions for various platforms, providing access to a multimodal exploration framework (Figure 2). This framework allows users to explore a logical dataset organization, perform semantic corpora queries, visualize 3D brain atlases, and visualize functional connectome. The 3D visualization is powered by VTK, displaying anatomical data, image post-processing results, and analysis and simulation outcomes (34).

An iterative development and integration approach, guided by continuous user feedback, ensures the advancement and combination of various components to achieve a comprehensive solution. By incorporating lesion segmentation, whole brain modeling, and non-invasive stimulation functionalities, BrainX3 embodies the concept of personalized medicine, facilitating individualized patient care and intervention decisions. The platform's intuitive and powerful capabilities make it a prime example of how cutting-edge neuroinformatics technology can revolutionize healthcare by tailoring treatments to each patient's unique needs (34, 46, 105–108).

The integration of AI-based models for lesion identification and whole brain models within the BrainX3 platform presents a revolutionary step in personalized medicine. The combination of these cutting-edge technologies opens up new horizons for understanding and treating neurological disorders at an individual level. By leveraging AI algorithms for lesion identification, BrainX3 empowers neuroscientists and clinicians to accurately detect and classify lesions, providing crucial insights into the structural damage caused by these abnormalities. This precise lesion identification is vital for tailoring treatment plans to each patient's specific needs, enhancing the potential for successful interventions.

Furthermore, the incorporation of whole brain models in BrainX3 enables the replication of patient-specific brain activity, going beyond static lesion identification to grasp the dynamic and interconnected nature of the brain. With access to longitudinal patient-specific data, BrainX3 allows the exploration of irregularities in large-scale brain dynamics, shedding light on complex neural interactions underlying neurological conditions. This comprehensive understanding of brain dynamics can profoundly impact diagnosis and prognosis, laying the foundation for more informed and personalized treatment decisions.

Moreover, the amalgamation of lesion identification and whole-brain modeling opens up the possibility of identifying potential targets for non-invasive neuromodulation techniques within BrainX3. By analyzing the patient's connectome and structural data, BrainX3 can be used to pinpoint specific brain regions affected by the lesion and propose nodes that could serve as targets for non-invasive interventions. This breakthrough has significant implications for developing tailored therapeutic approaches, such as transcranial magnetic stimulation (TMS), transcranial direct current stimulation (tDCS), and transcranial ultrasound stimulation (TUS). These techniques have demonstrated the potential to promote neural plasticity, modulate excitability and improve brain function in various neurological disorders. With BrainX3′s capabilities, clinicians can identify optimal targets for neuromodulation and use embedded models to test the effect of the perturbation on such targets, thus increasing the efficacy of treatment while minimizing potential side effects.

In conclusion, the integration of AI-based lesion identification, whole-brain modeling, and the identification of potential targets for non-invasive neuromodulation within BrainX3 represents a pivotal leap forward in personalized medicine. This synergy between advanced technologies empowers clinicians to create highly individualized treatment plans, taking into account the unique characteristics of each patient's brain. As BrainX3 continues to evolve and refine its functionalities, it has the potential to revolutionize patient care, offering unprecedented opportunities to advance the field of neuroscience and transform the landscape of personalized medicine.

## Data availability statement

The original contributions presented in the study are included in the article/supplementary material, further inquiries can be directed to the corresponding author.

## Author contributions

VS: Writing—original draft. FP: Writing—original draft, Writing—review & editing. PV: Supervision, Writing—review & editing.
